# Supplementary material for: A human subcortical network underlying social avoidance revealed by risky economic choices
Source: eLife. 2019 Jul 22;8:e45249. doi: 10.7554/eLife.45249 (PMC6703852; doi:10.7554/eLife.45249)
Supplement: Supplementary file 1. — Four tables displaying additional results: Supplementary file 1A and 1B show descriptive statistics for questionnaire data from Study one and Study two respectively, Supplementary file 1C and 1D show all steps of a backward stepwise regression analysis for the experiment data of Study one and Study 2, respectively. [file elife-45249-supp1.docx]

**Supplementary file**

Behavioural data: questionnaires

Descriptive statistics, internal consistency measures (Cronbach’s Alpha correlation values) and reliability measures (split-half correlation values) of the questionnaire data are shown for both studies in Tables S1 and S2 below.

**Supplementary file** **1A**: Questionnaire data from Study 1

|  |  | LSAS | AQ |  |
| --- | --- | --- | --- | --- |
| Valid |  | 68 | 68 |  |
| Missing |  | 0 | 0 |  |
| Mean |  | 28.81 | 14.57 |  |
| Std. Deviation |  | 24.43 | 5.511 |  |
| Minimum |  | 2 | 5 |  |
| Maximum |  | 118 | 35 |  |
| Cronbach’s Alpha |  | 0.96 | 0.60 |  |
| Split-half reliability |  | 0.97 | 0.50 |  |

**Supplementary file** **1B**: Questionnaire data from Study 2

|  |  | | LSAS | AQ | STAI-T |  | BDI |  |  |
| --- | --- | --- | --- | --- | --- | --- | --- | --- | --- |
| Valid |  | | 47 | 45 | 46 |  | 46 |  |  |
| Missing |  | | 0 | 2 | 1 |  | 1 |  |  |
| Mean |  | | 29.34 | 16.80 | 36.41 |  | 6.44 |  |  |
| Std. Deviation |  | | 22.97 | 6.58 | 11.18 |  | 8.06 |  |  |
| Minimum |  | | 0 | 8 | 21 |  | 0 |  |  |
| Maximum |  | | 86 | 39 | 71 |  | 31 |  |  |
| Cronbach’s Alpha | |  | 0.96 | 0.53 | 0.53 |  | 0.92 |  |  |
| Split-half reliability | |  | 0.91 | 0.46 | 0.55 |  | 0.90 |  |  |

Behavioural data: full backward stepwise regression models

**Supplementary file** **1C**: backward stepwise regression models for Study 1

| **Model** | |  | | **Unstand. coeff.** | | **Stand. error** | | **Standard. coeff.** | **t** | | **p** | |  |  |
| --- | --- | --- | --- | --- | --- | --- | --- | --- | --- | --- | --- | --- | --- | --- |
| 1 |  | (Intercept) |  | -0.105 |  | 0.204 |  |  |  | -0.515 |  | 0.608 | |  |
|  |  | LSAS |  | -0.010 |  | 0.003 |  | -0.469 |  | -2.989 |  | 0.004 | |  |
|  |  | AQ |  | 0.026 |  | 0.015 |  | 0.262 |  | 1.753 |  | 0.084 | |  |
|  |  | Gender |  | 0.033 |  | 0.139 |  | 0.030 |  | 0.238 |  | 0.812 | |  |
|  | | | | | | | | | | | | |  |  |

**Supplementary file** **1D**: backward stepwise regression models for Study 2

| **Model** | | |  | | **Unstand. coeff.** | **Stand. error** | | | **Standard. coeff.** | | **t** | | | **p** | | |  |  |
| --- | --- | --- | --- | --- | --- | --- | --- | --- | --- | --- | --- | --- | --- | --- | --- | --- | --- | --- |
| 1 |  | (Intercept) | |  | 0.025 |  | 0.164 |  | |  | |  | 0.150 | |  | 0.881 | |  |
|  |  | LSAS | |  | -0.007 |  | 0.002 |  | | -0.636 | |  | -3.392 | |  | 0.002 | |  |
|  |  | STAI_T | |  | 0.004 |  | 0.005 |  | | 0.206 | |  | 0.793 | |  | 0.432 | |  |
|  |  | AQ | |  | 0.003 |  | 0.005 |  | | 0.079 | |  | 0.544 | |  | 0.590 | |  |
|  |  | BDI | |  | -0.009 |  | 0.007 |  | | -0.315 | |  | -1.372 | |  | 0.178 | |  |
|  |  | Gender | |  | 0.094 |  | 0.074 |  | | 0.197 | |  | 1.268 | |  | 0.212 | |  |
|  | | | | | | | | | | | | | | | | |  |  |
